# Viral Resistance and IFN Signaling in STAT2 Knockout Fish Cells

**DOI:** 10.4049/jimmunol.1801376

**Published:** 2019-05-29

**Authors:** Carola E. Dehler, Katherine Lester, Giulia Della Pelle, Luc Jouneau, Armel Houel, Catherine Collins, Tatiana Dovgan, Radek Machat, Jun Zou, Pierre Boudinot, Samuel A. M. Martin, Bertrand Collet

**Affiliations:** * University of Aberdeen, AB24 2TZ Aberdeen, United Kingdom;; † Marine Scotland, Marine Laboratory, AB11 9DB Aberdeen, United Kingdom; and; ‡ Virologie et Immunologie Moléculaires, Institut National de la Recherche Agronomique, Université Paris-Saclay, 78352 Jouy-en-Josas cedex, France

## Abstract

IFN belong to a group of cytokines specialized in the immunity to viruses. Upon viral infection, type I IFN is produced and alters the transcriptome of responding cells through induction of a set of IFN stimulated genes (ISGs) with regulatory or antiviral function, resulting in a cellular antiviral state. Fish genomes have both type I IFN and type II IFN (IFN-γ), but no type III (λ) IFN has been identified. Their receptors are not simple counterparts of the mammalian type I/II IFN receptors, because alternative chains are used in type I IFN receptors. The mechanisms of the downstream signaling remain partly undefined. In mammals, members of the signal transducer and activator of family of transcription factors are responsible for the transmission of the signal from cytokine receptors, and STAT2 is required for type I but not type II IFN signaling. In fish, its role in IFN signaling in fish remains unclear. We isolated a Chinook salmon (*Oncorhynchus tshawytscha*) cell line, GS2, with a stat2 gene knocked out by CRISPR/Cas9 genome editing. In this cell line, the induction of ISGs by stimulation with a recombinant type I IFN is completely obliterated as evidenced by comparative RNA-seq analysis of the transcriptome of GS2 and its parental counterpart, EC. Despite a complete absence of ISGs induction, the GS2 cell line has a remarkable ability to resist to viral infections. Therefore, other STAT2-independent pathways may be induced by the viral infection, illustrating the robustness and redundancy of the innate antiviral defenses in fish.

## Key Points

- A *stat2*^−/−^ *Oncorhynchus tshawytscha* cell line was isolated.
- STAT2 is required for type I but not type II signaling in salmonid cells.

## Introduction

Interferon is a family of cytokines responsible for driving antiviral immune processes (1). Upon a viral infection, most nucleated cells produce type I IFN directing the early antiviral innate immune response. Specialized immune cells such as lymphocytes produce type II (γ-) IFN, initializing the adaptive immune response to viruses. Cells respond to IFN through a signaling process involving specific recognition and activation of type I, II, and III IFN by specific receptor complexes expressed at the cell surface: IFNAR1/2–Jak1–Tyk2, IFNGR1/2–Jak1/2, and IFNλR1/IL–10R2–Jak1/2–Tyk2, respectively (2, 3). The resulting conformational changes of the receptors create docking sites for key downstream signaling molecules, the STAT-1 or 2, that get phosphorylated at specific sites (Y701, S708, S727 for STAT1 and Y690 for STAT2).

The type I IFN receptor complex docks and phosphorylates both STAT1 and STAT2, which combine with IFN regulatory factor (IRF)–9 to form a heterotrimeric complex termed IFN stimulated gene (ISG) factor 3 (ISGF3) that translocates into the nucleus and activates IFN-stimulated response elements (ISRE) within the promoters of a set of ISGs. The ISG consists of a distinct panel of genes, often with direct antiviral or regulatory functions responsible for the establishment of an antiviral state that blocks viral replication and limits spread of the virus (4).

In contrast, the type II IFN receptor complex exclusively docks and phosphorylates STAT1, resulting in the formation of a STAT1/STAT1 homodimeric complex that translocates into the nucleus and activates IFN-γ activation site (GAS) elements in the genome resulting in the induction of a GAS-specific set of genes (5). In fish, functional ISRE and GAS have been identified in promoter of a discrete number of ISGs (6–8).

The genome of early teleost fish was duplicated 320–350 million years ago (9) whereas salmonid fish went through an additional genome duplication event ∼100 million years ago (10–13). Although many genes are lost after a whole genome duplication event, salmonid IFN and ISG repertoires are complex, with potentially four paralogous genes for each gene described in mammals (14, 15).

Typical genes coding for type I and type II, but not type III, IFN have been identified in fish genomes. In fact, IFN and many ISG genes have shown high rates of change during evolutionary time in fish as in tetrapods, and the number of paralogs can be greatly expanded or reduced to a single gene. Fish type I IFN genes were prone to extensive diversification, especially in Salmonids (16), as is also observed in mammalian genomes (17). Importantly, fish homologs of the key signaling factors downstream of IFNR, such as STAT1/2 and IRF9, have been found, indicating that the general structure of the pathways is probably similar in fish and mammals. However, several important differences between fish and mammalian’s IFN system hamper a straightforward comparison: 1) in fish, many signaling factors have several paralogs for a unique counterpart in human or in the mouse, which can lead to subfunctionalization. Thus, the multiple fish Jak paralogs are not employed equally for the transmission of the IFN-γ signal (18). 2) Fish type I IFN are divided in two main classes using different receptors, which have some features in common with components of the receptors for mammalian type III IFN (18–20), and (3) fish IFN-γ1 and IFN-γ2 do not share the same receptor either (18). Hence, the precise role of the different signaling factors downstream of IFN receptors remains unresolved in fish.

STAT proteins are no exceptions to this. They have been described as an ancient and highly conserved family, with most members already defined in the common ancestor of fish and mammals (15). Also, *stat1* and *stat2* genes are induced by viral infections in different fish species, suggesting that they are implicated in the antiviral response as their homologs in mammals. However, STATs show varying levels of paralogue retention in salmonids, with four *stat1* copies for only a single *stat2* copy. The respective roles of the different STAT1 and of STAT2 in IFN signaling is therefore still undefined.

In the current study, we produced a salmonid cell line in which *stat2* has been disrupted using a CRISPR/cas9 based approach. We used these cells to demonstrate that STAT2 is necessary for the type I but not type II IFN signaling pathway. The *stat2* mutation resulted in increased production of viral particles of the DNA virus epizootic hematopoietic necrosis virus (EHNV) and with the least extent of the RNA virus viral hemorrhagic septicemia virus (VHSV). However, the severe disruption of the type I IFN induced by the lack of functional STAT2 was not associated with viral hypersensitivity and fast, dramatic cell destruction.

## Materials and Methods

### Isolation of the GS2 cell line

**FIGURE 1.**
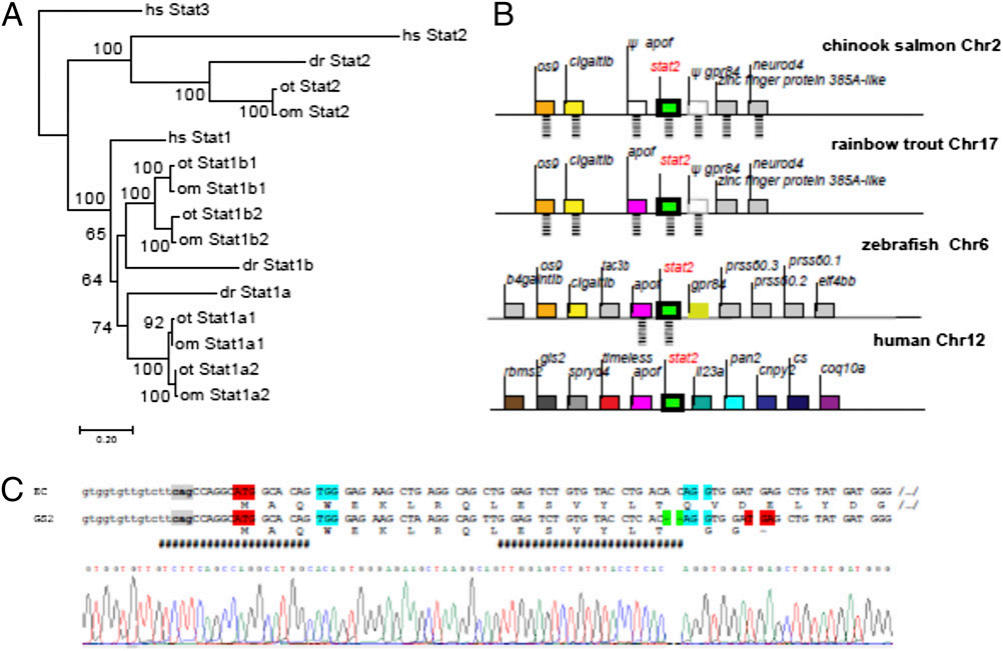
Genomic structure of the *stat1* and *stat2* loci in salmonid fish. (**A**) Phylogenetic tree of Stat1 and Stat2 in rainbow trout and Chinook salmon. The evolutionary history was inferred using the neighbor-joining method. The bootstrap consensus tree inferred from 500 replicates is taken to represent the evolutionary history of the taxa analyzed. Branches corresponding to partitions reproduced in <50% bootstrap replicates are collapsed. The percentage of replicate trees in which the associated taxa clustered together in the bootstrap test (500 replicates) are shown next to the branches. The evolutionary distances were computed using the JTT matrix-based method and are in the units of the number of amino acid substitutions per site. The rate variation among sites was modeled with a γ distribution (shape parameter = 1). (**B**) Synteny analysis of the *stat2* locus in Chinook salmon, rainbow trout, zebrafish, and human. (**C**) Location of the two sgRNA (represented in pink by ####) and chromatogram obtained from direct sequencing of purified PCR product amplified from genomic DNA purified from GS2 cells. The intronic and exonic sequences are in lower and upper case, respectively. The intron 1 acceptor site is in gray, and the protospacers are in cyan. The 2-nt deletion is highlighted in green, the start and premature stop codons are in red.

For generation of the *stat2* knockdown cell line, a Chinook salmon (*Oncorhynchus tshawytscha)* embryo (CHSE) cell line that was previously modified to stably express both a monomeric enhanced green fluorescence protein (mEGFP) and Cas9 (CHSE-EC) was chosen as the starting point of this study, further referred to as EC (21). The whole genomes of two different *Oncorhynchus* species (Chinook salmon *O. tshawytscha* and the rainbow trout *O. mykiss*) were examined for the presence and copy numbers of the *stat2* gene, which consistently showed as a single copy located on chromosome 2 and 17 of the Chinook salmon and rainbow trout genome, respectively (22; Fig. 1A, 1B). A small fragment containing the start of the *stat2* open reading frame (ORF) was amplified from genomic DNA purified from the EC cell line using the primers STAT2F and STAT2R, purified and sequenced (see Table I). Two single guide RNAs (sgRNAs) located in the first 50 nt of the *stat2* ORF (Fig. 1C) were produced by a combination of PCR and in vitro transcription. To identify potential off-targets of the guide RNA, both sgRNA1 and sgRNA2 sequences were used to search against nucleotide sequences using Blastn limited to highly similar sequences (megablast) and restricted to entries associated with the family “*Oncorhynchus*” (taxid: 8016). No off-target genes with 100% coverage and 100% identity were identified for either sgRNA1 or sgRNA2, suggesting high specificity of the designed guide RNAs. To generate the sgRNA template, amplification of a 120 nt blunt-ended PCR product was carried out using the Q5 Taq polymerase (New England Biolabs). Each sgRNA template was amplified in five 50-μl reactions each with 25 μl 2× Q5 master mix, 400 nM DR274F forward primer (Table I), 40 nM STAT2R1-2 reverse primer, and 20 nM template STAT2T1-2 (see Table I). Cycling was as follows: 98°C for 30 s then 35 cycles of 98°C for 5 s, 60°C for 10 s, 72°C for 10 s, and a final extension of 72°C 30 s. The five reactions were pooled and purified using QIAquick PCR purification kit (Qiagen) according to the manufacturer’s instructions and eluted in 50 μl water. The sgRNAs were synthesized using the RiboMAX Express T7 kit (Promega) purified using TRIzol (Thermo Fisher Scientific) according to the manufacturers’ instructions. The sgRNAs were resuspended in RNAse- and DNAse-free water and quantified by nanodrop prior to transfection. The quality and purity of the RNA was verified on agarose–EtBr gel before or after RNase A treatment (Qiagen).

For transfection, the two *stat2* sgRNAs were mixed with the sgRNA targeting mEGFP and used to transfect EC cells; 100 ng of each of the three sgRNA (two targeting *stat2* and one *egfp*) per 10 μl of cell suspension was used as described previously (21). Transfected cells were plated onto a 75 cm^2^ flask and passaged weekly for 3 wk. Nonfluorescent single trypsinized cells were then individualized by FACS onto a 48-well tissue culture plate by a BD Influx BSLII Sorter (Iain Fraser Cytometry Centre, University of Aberdeen) and propagated in 1 ml culture medium for 1 mo. Four nonfluorescent clones (i.e., with mutated EGFP) were propagated in 25 cm^2^ flasks and characterized further.

Genomic DNA was purified by the HMW DNA kit and magnet (Qiagen) according to the manufacturer’s instructions. A 306-bp segment containing the targeted site was amplified by PCR from the genomic DNA using primers STAT2F and STAT2R (Table I). The PCR product was purified using QIAquick PCR purification kit (Qiagen) and directly sequenced using the same amplification primers (Sequencing service, University of Dundee).

All cells were grown at 22°C in EMEM medium supplemented with 500 μg/ml G418 (Sigma-Aldrich), 30 μg/ml Hygromycin (Thermo Fisher scientific), and 10% FBS (Nalgene).

### Characterization of EC and GS2 cell lines by quantitative RT-PCR

For the initial characterization of the response to IFN, EC and GS2 cells were seeded into six-well plates (Greiner Bio-One). Wells were left unstimulated or stimulated with 250 ng/ml recombinant rainbow trout type I IFN [IFNA2; AJ582754; (23)] or 250 ng/ml rainbow trout type II IFN [IFNG1; CAE82300; (23)] for 30 h. Recombinant IFNA2 and IFNG1 were produced as described previously (16, 23, respectively). Briefly, the recombinant type I and II IFN were HIS_6_-tagged versions of the rainbow trout IFNA2 and IFNG1, respectively, produced under native condition in *Escherichia coli* and purified under native conditions using NiTA columns. Following the stimulation, cells were lysed by addition of 1 ml of TRIzol (Thermo Fisher Scientific), and the total RNA was purified according to the manufacturer’s instructions. cDNA was synthesized from 500 ng of total RNA using the ABI MultiScribe reverse transcriptase with random hexamers priming according to the manufacturer’s instructions (Thermo Fisher Scientific). Gene expression was assessed by Quantitative RT-PCR (QPCR) using an LC480 real-time PCR thermocycler (Roche) with OneTaq Hot start DNA polymerase 2× mastermix (New England Biolabs) and 50× SYBR Green (Thermo Fisher Scientific). Primers were designed across splicing sites to exclude amplification of genomic DNA (Table II) and verified by postamplification analysis of the melting properties. Several QPCR products were sequenced to verify the specificity of the assays. The elongation factor 1 α (*elf1a*) was used a PCR calibrator (see Table II), and the constitutive expression of this gene upon stimulation with type I IFN was verified by the analysis of the RNA sequencing (RNA-seq) data.

The full ORF of *O. mykiss stat2* gene (XP_021424957; *O. tshawytscha* genome not available at the time) was obtained by gene synthesis (GeneArt Gene Synthesis Service; Thermo Fisher Scientific) and subcloned into the pcDNA3.1-Hyg expression vector between *Nhe*I and *Pme*I sites to obtain an expression plasmid, pS2, expressing STAT2. A control plasmid pcDNA3.1-Hyg-mEGFP (pG; 21) was used as a control plasmid. GS2 cells were transiently transfected with pS2 or pG according to (21) and seeded into six-well plates for 30 h. For each group, three wells were stimulated with rIFNA2 250 ng/ml and three wells were left untreated for 30 h, following which the cells were harvested in 600 μl of RLT buffer (Qiagen) with 1% v/v 2-ME (Sigma-Aldrich) and stored at −80°C until processed. Lysate was homogenized using QIAshredder (Qiagen), and total RNA was purified with RNeasy mini kit (Qiagen) according to the manufacturer’s instructions. QPCR analysis was carried out as described above.

In a second experiment, GS2 cells were cotransfected as described above with a transfection control plasmid (pRFP-KDEL), pmx-EGFP expressing EGFP under the control of the rainbow trout Mx1 gene promoter obtained by *Sac*I/*Xho*I-subcloning from pGL3-Neo-Basic-pomMx1 (24; Addgene no. 30536) into pGL4.22-Pur vector (Promega), pS2, or a control plasmid pcDNA3.1-Hyg (pCont; Thermo Fisher Scientific). Transfected cells were plated on a ibidi multiwell slide chamber and stimulated with rIFNA2 for 40 h at 20°C, and the EGFP and RFP fluorescence were visualized on a Zeiss fluorescent microscope.

### Viral infection

The viral isolates used were salmon pancreatic disease virus (SPDV) isolate F07-220, VHSV (25), and EHNV (25, 26).

For assessment of viral infection by cytopathogenic effect, EC and GS2 cells were seeded on the same 96-well plate (200 μl cell suspension per well). Four wells were left uninfected, and for each cell line a series of four wells were infected with serial dilutions (1–100,000) of inoculum. The viral titer was identical for all three viral isolates and estimated to 10^2^ PFU/ml, corresponding to a multiplicity of infection (m.o.i.) of ∼0.01 for the lowest dilution. The procedure was carried out in three sets of plates and harvested after 3, 5, and 7 d postinfection (dpi). The quantification of cytopathogenic effect was performed as described previously (25). Briefly, dilutions of inoculum were incubated for different durations, the cells were fixed, stained with crystal violet, and redissolved, and the OD at 450 nm was read. The percentage of viral cytopathology was calculated as $100*\left(\frac{OD_{c}-OD_{v}}{OD_{c}}\right)$, with *OD_c_* as OD of uninfected cells and *OD_v_* as OD of infected cells.

The release of viral particles over time was measured by infecting the EC and GS2 cell lines grown in 25 cm^2^ flask at the same density with EHNV (3.1 × 10^5^ PFU/ml ∼m.o.i. of 0.3) or VHSV (1.1 × 10^6^ PFU/ml ∼ m.o.i. of 0.1) and collecting supernatant after 1, 2, and 5 d. Titers of infectious virions were measured by plaque assay on monolayers of Epithelioma papulosum cyprini cells (EPC; ATCC CRL-2872). Collected supernatants were serially diluted in duplicates for the plaque assay. The infection was performed at 14°C under a layer of methylcellulose (0.75% final concentration) for 3 d after an adsorption step at 14°C for 1 h in liquid phase. The plaques were fixed with formaldehyde (10%), stained using crystal violet (1% final dilution), and photographed.

### Transcriptome deep sequencing analysis

#### IFN stimulation.

For IFNA2 stimulations, two 75-cm^2^ flasks each for EC and GS2 were grown to full confluency as described above. The cells were then rinsed with Dulbecco’s PBS twice and detached with 4 ml of trypsin per flask. The cells of the replicate flasks for each cell type where combined in a falcon tube with 2 ml of Eagle’s MEM (EMEM) medium (10% FBS) and suspended. One milliliter of each cell mix was seeded in a 25 cm^2^ flask to create 10 replicate flasks for each cell type. The cells were grown to 80% confluency over 3 d at 22°C, after which all cells were rinsed with Dulbecco’s PBS and the excess was pipetted off. For IFNA2 stimulation, EC and GS2 cells in five replicate flasks were incubated in 25 ml of EMEM medium supplemented with 250 ng/μl of INFA2 (stock 30 μg/ml), and a further five flasks of each cell type were designated as control cells and had 5 ml of fresh EMEM added. All flasks were incubated for 30 h, after which RNA was extracted as described in *Characterization of EC and GS2 cell lines by quantitative RT-PCR*.

#### Illumina library preparation and sequencing.

For each of the four experimental groups, three biological replicates were used for library construction. The 12 RNA-Seq libraries were prepared using TruSeq Stranded mRNA Sample Preparation Kit (Illumina) according to the manufacturer’s instructions. Briefly, Poly-A RNA were purified from 500 ng of total RNA with External RNA Controls Consortium as a control using oligo(dT) magnetic beads, fragmented, and retrotranscribed using random primers. Complementary DNAs were end-repaired and 3-adenylated; indexed adapters were then ligated. Fifteen (15) cycles of PCR amplification were performed, and the PCR products were cleaned up with AMPure beads (Beckman Coulter). Libraries were validated for quality on Agilent DNA1000 Kit and quantified with the QPCR NGS Library Quantification kit (Roche). The final libraries were pooled in equimolar amounts and sequenced in pair-ends 2 × 75 bp on Illumina NextSeq 500/550 High Output v2 kit. For each library, a depth 20 M reads were generated.

#### Mapping reads and gene expression counts.

The read quality was checked with FastQC in the ng6 environment (27). Reads were then spliced-aligned to 47,898 genes (47,022 Gnomon, 876 RefSeq, GCF_002163495.1_Omyk_1.0_genomic.gff from the National Center for Biotechnology Information [NCBI]) with two additional chromosomes harboring sequences of the pmEGFP-N1 and pcDNA3.1-Hyg-nCas9n plasmids (28) using TopHat v2.0.14 software (29; parameters -N 2 -read-edit-dist 2–b2-sensitive–no-coverage-search). The average number of mapped read per sample (R1 + R2) was 5.27 million ± 0.5. Only fragments mapping coherently and unambiguously have been considered for gene counts. Gene counts have been assigned using featureCounts v1.5.2 (30).

### Identification of differentially expressed genes

Differentially expressed genes (DEGs) between cells treated with IFNA2 and controls were identified for EC and GS2 cell lines. Additional comparison between EC control and GS2 control was carried out to inform on the differential basal levels of expression between the two cell lines. DEGs were identified using DESeq2 1.6.3 (Bioconductor) (31) and R-3.4.2 (27). Briefly, raw counts of genes were subjected to a minimal prefiltering step; genes for which the count sum, per group of samples, was equal or higher than 10 in at least one group, were kept. Raw counts were normalized for library size, and normalized data were fitted using a negative binomial general linear model. Data were adjusted for multiple testing using the Benjamini–Hochberg procedure (adjusted *p* value). Genes with an adjusted *p* value < 0.01 and an absolute fold change (FC) > 2.5 or FC < 0.4 were considered as DEGs.

#### Functional annotation analysis.

For each rainbow trout gene (assembly Omyk_1.0, NCBI), the longest protein model was extracted and used as a bait for blastp analysis against the human proteome (Ab initio, Ensembl GRCh38). The best blast hit annotations were collected (*e* value <0.01).

#### Statistical analyses.

QPCR data analysis was carried out as described previously (23). The comparisons between experimental groups in the gene expression levels were carried out by *t* test on the log-transformed levels of expression relative to *elf1*a gene, followed by Bonferroni correction for multiple comparison. The comparison between viral cytopathology levels were carried out in a similar way after logit transformation. All variables were considered heteroscedastic.

## Results

### The genome of Chinook salmon contains a single stat2 gene

TBlastn analysis of the genome assembly of *O. tshawytscha* identified a unique sequence highly similar to human *stat2* gene, corresponding to the locus LOC112217577 on chromosome 2. This gene encodes a protein (814 AA, XP_024233747) that is 42% similar to the human STAT2 (851 AA). Single *stat2* genes were found in the other fish genomes, including other salmonids belonging to the genus *Oncorhynchus* and *Salmo*. These included Atlantic salmon (*Salmo salar*), rainbow trout (*O. mykiss*), coho salmon (*O. kisutch*), and Chinook salmon (*O. tshawytscha*), from which derives the EC line, expressing nCas9n and mEGFP constitutively (21). Only in Atlantic salmon (*S. salar*) have two *stat2* sequences been reported (32), but only one gene is present in the current genome assembly. Hence, only one copy of *stat2* has been retained in salmonids after the two whole genome duplications that occurred during the early evolution of teleosts and salmonids, respectively. All *stat2* sequences from fish and tetrapods cluster in a well-supported branch of the phylogenetic tree (Fig. 1A) that recapitulates the tree of species, suggesting that *stat2* sequences constitute a well-supported group of orthologs. Other fish *stat* sequences are not found in this branch and group with their human respective counterparts, indicating that the *stat* family had already diversified in the common ancestor to fishes and mammals in a similar way as *irf* or *socs* gene families (15). In keeping with this, Chinook salmon and rainbow trout *stat2* genes belong to synteny groups partly conserved in zebrafish and human, which confirms the orthology shown by the tree (Fig. 1B). Of note, no more additional *stat2*-like sequences could be found in salmonids from the genus *Oncorhynchus*, either from Whole Genome Shotgun or from Expressed Sequence Tags databases, strongly suggesting that the genome of this genus, indeed contain only one copy of this gene.

### Production of GS2, a double mutant stat2^−/−^ EC cell line

Following stat2- and egfp- sgRNA transfections, four nonfluorescent clones (i.e., mutants in *egfp*) were chosen for characterization, propagated, and characterized. One clone was not mutated in *stat2*, one had a large deletion of 42 nt (without any frame shift), one had a silent single nucleotide substitution, and one had a 2-nt deletion leading to a frame shift with all mutations found at the expected site; this final clone was termed GS2. The frame shift mutation was biallelic and homozygous; the fact that the two chromatids were carrying the same mutation was confirmed by the purity of the Sanger trace from direct sequencing of the purified PCR product (Fig. 1C). The mutated ORF of the GS2 cells encodes for an 18-aa peptide, with the first 16 residues matching the original N-terminal of the STAT2 protein (Fig. 1C), which implies that the STAT2 functions are fully disrupted in mutant cells (Tables I–III).

**Table I.** Primers used for the characterization of the targeted site and for the synthesis of sgRNAs

Name	Sequence	Use
STAT2F	5′-GTGGTCAGACCACTGGCAGC-3′	Sequencing sgRNAs stat2 locus
STAT2R	5′-GGATGAGTGGACTGCCTCACC-3′	
STAT2T1	5′-AAAAGCACCGACTCGGTGCCACTTTTTCAAGTTGATAACGGACTAGCCTT ATTTTAACTTGCTATTTCTAGCTCTAAAACCTGTGCCATGCCTGGCTGAA-3′	Production of the stat2 sgRNA1
STAT2R1	5′-AGCTAATACGACTCACTATATTCAGCCAGGCATGGCACAG-3′	
STAT2T2	5′-AAAAGCACCGACTCGGTGCCACTTTTTCAAGTTGATAACGGACTAGCCTT ATTTTAACTTGCTATTTCTAGCTCTAAAACGTGTCAGGTACACAGACTCC-3′	Production of the stat2 sgRNA2
STAT2R2	5′-AGCTAATACGACTCACTATAGGAGTCTGTGTACCTGACAC-3′	
DR274F	5′-AAAAGCACCGACTCGGTGCCAC-3′	Production of stat1 and stat2 sgRNAs

The target for sgRNAs are underlined.

**Table II.** Primers used for QPCR gene expression

Gene	Sequence 5′-3′ Forward	Sequence 5′-3′ Reverse	Accession Number
*mx*	5′-GATGCTGCACCTCAAGTCCTATTA-3′	5′-CGGATCACCATGGGAATCTGA-3′	*mx1* XM_024415949; *mx3* XM_024415945
*pkr*	5′-GAAAACCTTCACTCTGAGGG-3′	5′-GACATGAAACCGATGCATCC-3′	XM_024425247
*dhx58*	5′-GCTGGTCAACAAGGTTCATTTGGTTG-3′	5′-GCAGAGTGAACTGTGAGAGC-3′	XM_024390155
*irf1*	5′-TTCTACACATCTTTCCAAGTGTCA-3′	5′-GGGTTTCTTGGTGACTGTCTT-3′	XM_024432485
*ifna3*	5′-ACTGAAACGCTACTTCAAGAAGTTGA-3′	5′-GCAGATTATGTTTCGTCTCTTTCCT-3′	XM_024434108
*stat2*	5′-CCCCACCGGTGAGCCTGATG-3′	5′-GACTATCCGCTCCACTCTTCT-3′	XM_024377979
*elf1a*	5′-CCCCTCCAGGATGTTTACAAA-3′	5′-CACACGGCCCACGGGTACA-3′	XM_024441752

**Table III.** Summary of the levels of expression and induction of the stat1 and stat2 genes as measured by RNA-seq in EC and GS2 following stimulation with IFNA2

	Accession Number (GeneID NCBI)		Adjusted *p* Value (FC)		
Name	*O. mykiss*	*O. tshawytscha*	EC ifn, EC control	GS2 ifn, GS2 control	EC control, GS2 control
stat 1a1	NP_001118179 (GeneID 100136755*^a^*)	LOC112266551*^b^*, LOC112253955*^b^*	NS	NS	NS
stat 1a2	XP_021463912 (GeneID 100137016*^a^*)	LOC112253897*^b^*, LOC112253778*^b^*	NS	NS	NS
stat 1b1	XP_021434868 (LOC110501544)	LOC112244575	<0.0001 (10.6)	NS	NS
stat 1b2	XP_021452654 (LOC110520020)	LOC112235369	NS	NS	NS
stat 2	XP_021424956 (LOC110494323)	LOC112217577	<0.0001 (2.1)	NS	<0.0001 (1.8)

^a^ Provisional RefSeq status as of 13th March 2019.

^b^ Duplication due to assembly errors in the *O. tshawytscha* genome as of March 13, 2019.

### Disruption of stat2 abolished the cell response to type I, but not II, IFN

**FIGURE 2.**
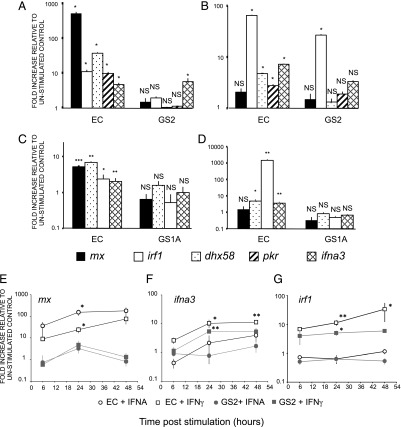
Gene expression in EC and GS2 cell line after IFNA2 or IFNG1 stimulation. Expression levels of ISGs *mx*, *irf1*, *dhx58*, and *ifna3* ISGs measured by QPCR in EC and GS2 cell lines after 30 h induction with recombinant rainbow trout type I (IFNA2) (**A**) or type II (IFNG1) (**B**) IFN. Expression levels of ISGs *mx*, *irf1*, *dhx58*, and *ifna3* ISGs measured by QPCR in EC and GS1A cell lines after 30 h induction with recombinant rainbow trout type 1 (IFNA2) (**C**) or type II (IFNG1) (**D**) IFN. The GS1A cell line has been obtained using a similar approach than GS2 (Supplemental Fig. 1) * indicates level of significance of the fold increase versus corresponding control. Kinetics of induction of *mx* (**E**), *irf1* (**F**), and *ifna3* (**G**) genes in EC or GS2 cells after 6, 24, or 48 h stimulation with IFNA2 (IFNA) or IFNG1 (IFN-γ). Data represent average FC (*n* = 3) relative to the corresponding unstimulated control.

The expression of a number of key ISGs (*mx*, *irf1*, *dhx58*, *pkr*, and *ifna3*) were measured by real-time QPCR following stimulation with IFNA2 (Fig. 2A) or IFNG1 (Fig. 2B). After stimulation with the type I IFN IFNA2, a typical response of these typical ISGs was observed in the EC cells but was abolished in the GS2 cells, as shown by the lack of significant induction of *mx*, *irf1*, *dhx58*, and *pkr* genes (Fig. 2A). After stimulation with type II IFN (IFNG1), the induction profile in EC cells was different, with no significant induction of *mx* gene but a very potent and significant induction of *irf1* gene (Fig. 2B). Interestingly, *ifna3* was also significantly induced by IFNG1, in addition to *dhx58* and *pkr* (Fig. 2B). In GS2 cells, the induction level of all tested genes but *mx* remained significant, but was somewhat reduced compared with EC, suggesting a possible role of STAT2 in their expression. Importantly, the induction of *irf1* remained very high in GS2, with an FC of 26.8. Stimulated levels of irf1 induction were not significantly different between EC and GS2. Overall, our data indicate that the induction of typical ISGs by type I IFN (IFNA2) in EC cells is abolished in GS2 cells, whereas the response to type II IFN (IFNG1) remains significant in both cell lines.

Additionally, using a *stat1a1*^−/−^ *stat1a2*^−/−^ cell line (GS1A) obtained using the same procedure than GS2 (Supplemental Fig. 1), we showed that these mutations abolished all upregulation of *mx*, *irf1*, *dhx58*, and *ifna3* genes either by IFNA2 or by IFNG1 (Fig. 2C, 2D). Importantly, although *irf1* was significantly induced (1462 ± 269-fold; *p* < 0.01) in the EC cell line or in the GS2 line, it was not upregulated in GS1A cells (0.5 ± 0.1-fold; *p* = 0.42; Fig. 2D), indicating that the response to type II IFN was also affected in this case.

These conclusions based on gene expression in GS2 cells 30 h poststimulation with IFNA2 or IFNG1 were confirmed on a time course of induction 6, 24, and 48 h after addition of rIFNA2 or IFNG1 (Fig. 2E–G). Only *irf1*—but not *mx* nor *ifna3*—was significantly induced in the GS2 cell line after IFNG1 stimulation for 6, 24, or 48 h, corroborating the abolishment of IFNA2 but not IFNG1 signaling in the stat2^−/−^ cells and the *irf1* status as a marker for type II IFN activity.

**FIGURE 3.**
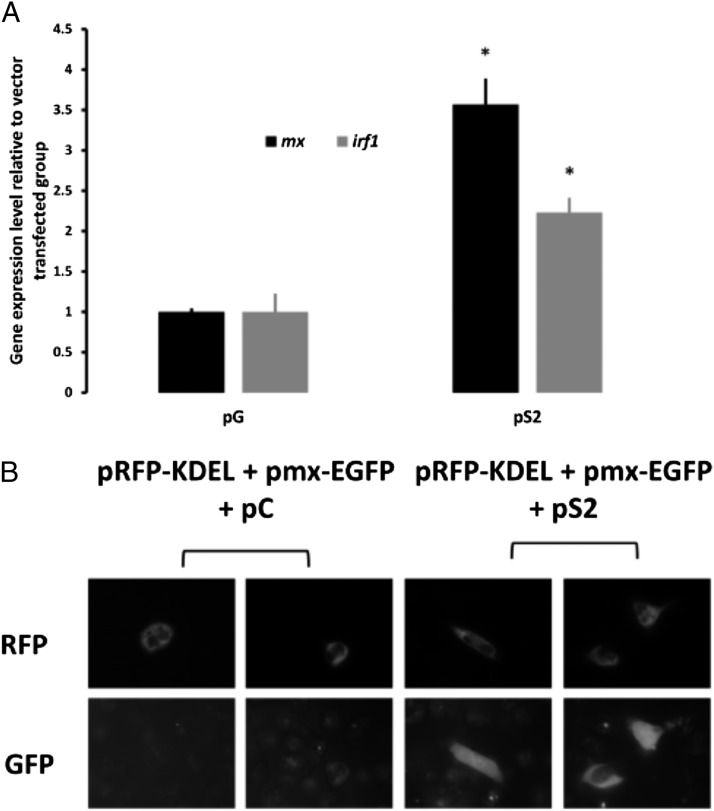
Restoration of STAT2 function in GS2 cells. (**A**) Gene expression levels of *mx* and *irf1* in GS2 cells that were transfected with plasmids overexpressing either mEGFP (pG) or Stat2 (pS2) after 24 h. The results are presented related to the pG group and therefore show the fold increase (*p < 0.05, *n* = 3). (**B**) Fluorescence of GS2 cells cotransfected with pRFP-KDEL (transfection control), pmx-EGFP (reporter for IFN activation), pS2 (rescue plasmid expressing STAT2), or pCont (empty vector) after 40 h stimulation with rIFNA2.

To verify that the modifications in IFN signaling are due to the mutation in *stat-2*, we transfected GS2 cells with a *stat2* expression plasmid and tested the effect of 30 h IFNA2 stimulation. As shown in Fig. 3A, the induction was 3.6- and 2.2-fold for *mx* and *irf1* genes, respectively, showing a significant restoration of Stat2 function. This was verified at the cellular level by the activation of a reporter system in transfected cells when STAT2 was provided by an expression plasmid after stimulation with rIFNA2. In transfected cells (positive for RFP), GFP was higher than the autofluorescence background only when GS2 cells were cotransfected with the pS2 plasmid expressing STAT2, demonstrating the restoration of the IFNA2 signaling pathway (Fig. 3B).

### Whole transcriptome analysis shows that the response to type I IFN (IFNA2) is almost fully abolished in GS2 cells

**FIGURE 4.**
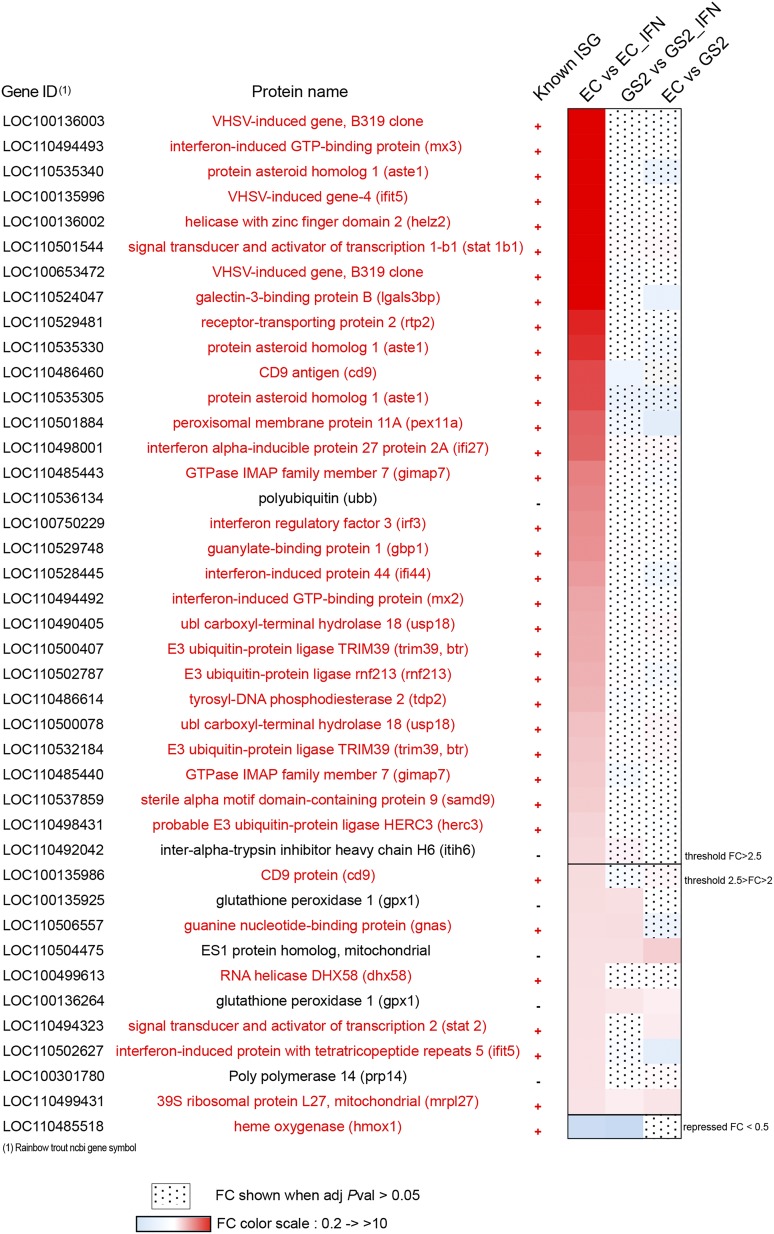
Whole transcriptome analysis of EC and GS2 cells after IFNA2 stimulation. List of the most inducible genes in the EC cell line after incubation with 30 h IFNA2 as determined by whole transcriptome sequencing. The heatmap represents the FC between control and induced conditions for the EC cell lines or the GS2 cell line, or between EC control and GS2 control cells. A complete list of genes significantly modulated by IFNA2 in EC and GS2 cells is provided in Supplemental Table I.

To achieve a global overview of the transcriptional response between EC and GS2 cells, the whole transcriptome was analyzed by Illumina deep sequencing (RNA-seq) with or without stimulation for 24 h with IFNA2. The dataset has been submitted to NCBI under the BioProject 495492. The RNA-seq mapping showed 30,536 unique loci were identified when aligned to the rainbow trout genome; of these, 27,972 were annotated as producing a functional protein. Expression changes of the 40 genes most induced by IFNA2 in EC are represented as a heatmap in Fig. 4, with 35 of them (88%) present in the Interferome database (33). None of these genes were found significantly induced in GS2 (Fig. 4), indicating a general breakdown of the ISG response to IFNA2. Genes upregulated by IFNA2 with an FC >5 in EC, but not in GS2, included several typical and well-conserved ISG such as *vtcn1*, *mx3*, *ifit5*, *helz2*, *stat1b1*, *cd9*, and *ifi27* (4, 15). A full table of genes significantly modulated by IFNA2 is shown in Supplemental Table I. In total, 34 genes were significantly induced more than 2.5-fold in the parental cells (EC) (51 genes more than 2-fold) when stimulated with IFNA2, whereas only a single gene was downregulated <0.4-fold (Supplemental Fig. 2, Supplemental Table I). In contrast, only two genes were significantly increased >2.5-fold in GS2 cells upon IFNA2 stimulation (44 genes more than 2-fold), with four genes decreased in expression <0.4-fold (16 <0.5-fold). A logFC/logFC representation of the differential response of EC and GS2 cells is shown in Supplemental Fig. 2A, 2B, highlighting the lack of highly induced genes in GS2. To further characterize the responses of EC and GS2 cells, we used both gene ontology (biological process) enrichment analysis and KEGG pathway analysis to identify the main functional groups that were enriched upon IFNA2 stimulation (Supplemental Fig. 2C). Results from this analysis showed a clear difference between the cell lines. The enrichment of biological processes in EC cells was significant for the Gene Ontology (GO) identifier “defense response to virus” (Benjamini corrected *e* value <5%), whereas the GO enriched biological process in GS2 was “Platelet aggregation” with the KEGG pathway “Regulation of actin cytoskeleton,” which did not evoke a link with the IFN-based antiviral response.

Interestingly, there were 224 (up) 178 (down) genes significantly altered between nonstimulated EC and nonstimulated GS2 cells (Supplemental Table II), suggesting that changes are induced by the Stat2 knockdown; however, these were not significantly enriched for GO functional terms related to IFN or antiviral activity.

The level of expression of *stat1* and *stat2* as measured by deep sequencing showed that only the *stat1b1* paralogue and *stat2* were found upregulated in EC, and neither in GS2, corresponding to the 6th and 37th genes in Fig. 4, respectively.

For further examination of the RNA-seq data, confirmation of the expression of the genes encoded by the stable transfected plasmids was made. The transcripts count mapped to the Cas9 expression plasmid was 42,387 and 18,437 for EC and GS2, respectively. The counts of transcripts mapped to mEGFP expression plasmid was 192,847 and 13 for EC and GS2, respectively, indicating a 99.99% reduction in the abundance of mEGFP transcripts in the GS2 cells related to EC. The transcript count for *elf1a*, which we used as house-keeping gene for QPCR, was 72,016 and 76,357 for EC and GS2, respectively. Interestingly, the mEGFP transcript count was the highest recorded value for any of the 30,535 mapped transcripts in any of the samples analyzed and may be a contributor to the transcriptional activity differences between the EC and GS2 control cells.

### Moderate modification of the sensitivity of GS2 cells to viral infection

**FIGURE 5.**
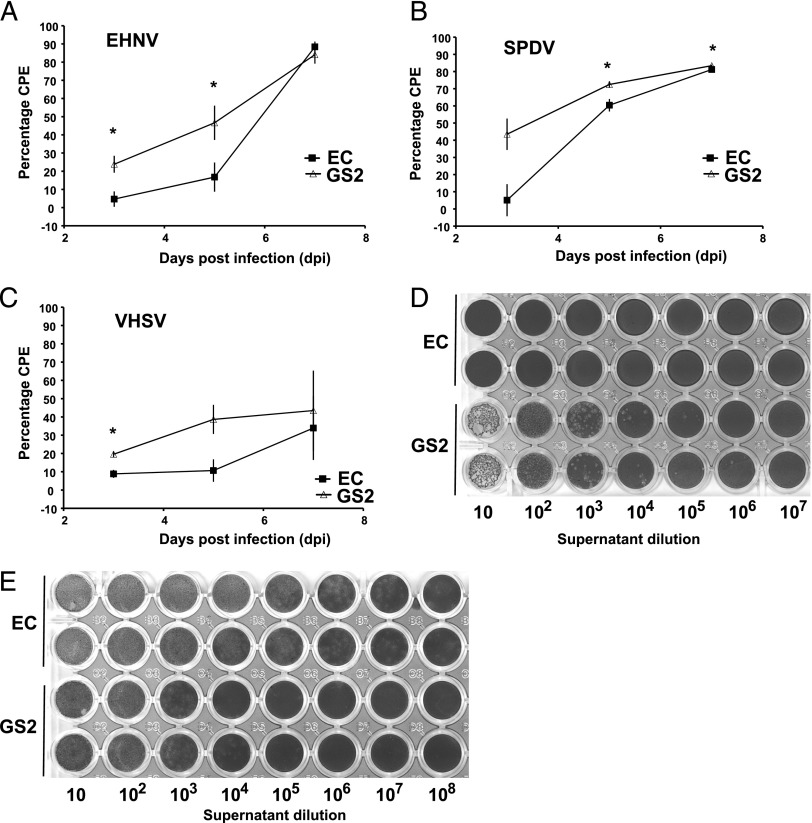
Percentage of viral cytopathogenicity after 3-, 5-, or 7-d infection and viral titer in EC and GS2 cell lines. Viral cytopathology measured postinfection with EHNV (**A**), SPDV (**B**), and VHSV (**C**). Visualization of the viral titer determined on EPC cells in supernatant from EC or GS2 cells infected with EHNV for 2 d (**D**) or VHSV for 5 d (**E**).

For SPDV, EHNV, and VHSV, the cytopathic effect was significantly higher in GS2 than in EC cells; for SPDV this was observed at 5 and 7 dpi, for EHNV at 3 and 5 dpi, and VHSV was only increased at 3 dpi (Fig. 5A–C). At 7 dpi, the maximum increase in percentage of cytopathology in GS2 was for VHSV, with 33.9 ± 17.5% in EC and 43.5 ± 21.9% in GS2, but the difference was not significant. At 7 dpi, the only consistent significant, but minimal, increase was for SPDV, for which the percentage cytopathology was 81.2 ± 1.5% in EC and 83.4 ± 0.7% in GS2.

The titrations of EHNV at 2 dpi and VHSV at 5 dpi from EC and GS2 supernatants are shown in Fig. 5D, 5E, respectively. Clear signs of EHNV infection with visible plaques were seen for the GS2 cell line up to dilution 10^4^–10^5^, but no signs were visible for the EC cell line supernatant at any dilution (Fig. 5D). Similar results were seen with supernatant collected at dpi 1 and 2 (data not shown). No clear differences could be observed in the viral titer of supernatant collected after VHSV infection with signs of infection in both cell lines (Fig. 5E).

## Discussion

Type I IFN are central to the host defense against viruses and are believed to activate via a conserved Jak/Stat signaling pathway. As part of our effort to unravel the detailed IFN signaling mechanisms in cells from a genome-duplicated lower vertebrate, we demonstrate for the first time, to our knowledge, that Stat2 is an obligatory player in type I IFN signaling, confirming that this pathway is conserved between teleosts and mammals.

### Workflow for salmonid knockout cell lines

The present work demonstrates a robust approach to generate a knockout somatic salmonid fish cell line with a high efficiency by using a fluorescent reporter transgene as a screening cotarget (21). The parental cell line, EC, constitutively expressing a functional nuclear nCas9n and monomeric mEGFP, is transiently transfected with a mix of sgRNAs designed against the gene of interest (*stat2* in this study) and against *megfp*. mEGFP-deficient single cells are isolated by FACS and left to propagate into clonal cell lines. Because of the slow growth of fish cells, the timeline for the isolation of such a cell line was ∼3 mo from the initial sgRNA transfection to the isolation of clonal cell lines, with sufficient material to characterize its genotype and its phenotype by PCR sequencing and QPCR, respectively.

The target gene *stat2* was present as a single copy in the genomes of rainbow trout, coho salmon, and Chinook salmon. The current assembly of the Atlantic salmon *S. salar* genome also contains a single copy of the *stat2* gene. From the RNA-seq data, only one *stat2* transcript could be detected in the EC cell line with or without IFNA2 induction, confirming the in silico analyses. In contrast, there were four paralogs of *stat1*. Two copies were closely related to the zebrafish *stat1a* gene that was not induced by Chikungunya infection, and two copies corresponding to *stat1b*, which was significantly upregulated (15). In the current study, out of the four mEGFP-deficient clones isolated, and three were mutated in the targeted gene *stat2*, with one of them being a null mutant. Besides the successful multiplex gene editing, it is remarkable that all three clones isolated were carrying homozygote biallelic mutations on the *stat2* locus similarly to results obtained in previous studies in mouse embryonic stem cells (34, 35) or in embryos of other fish species or in agnathans [Zebrafish (36); Lamprey (37); Medaka (38); Salmon (39)]. In somatic cells, the exact mechanism by which Cas9 induces homozygote biallelic mutation is currently unknown. It is important to have in mind that the cotargeted locus, *megfp*, in the EC cell line is a transgene with an uncharacterized site(s) of genomic integration.

The IFNA2 inducibility was partially restored by transfection of GS2 cells with a STAT2 expression plasmid. The rescue of stat2 function was carried out by transfection with an expression plasmid. Because fish cell lines are traditionally difficult to transfect, only a small percentage of GS2 cells had their STAT2 function restored, and the vast majority of cell were untransfected and still deficient in IFN signaling. By cotransfection with a reporter system, the GS2 rescue could be visualized at the cellular level when STAT2 was expressed and IFN present.

### Strong nonsense-mediated mRNA decay for egfp but not stat2 in GS2 cell line

The expression level of the *stat2* mRNA was not affected by the null mutation, suggesting that its transcript is resistant to the nonsense-mediated mRNA decay (NMD). This was expected, as in the corresponding mutated *stat2* mRNA the premature termination codon is close to the 5′ untranslated region and would escape NMD destruction (40, 41). In contrast, there was a 99.99% decrease in the *megfp* transcript abundance in the GS2 relative to EC cells. This result is surprising, as the mEGFP transgene is intronless (21) and as such, according to some NMD models, is thought to be immune to the NMD pathway (42). In this context, the marked difference in the NMD sensitivity of the *stat2* and *egfp* transcripts in GS2 cells is interesting and may suggest an alternative NMD model (43) or a peculiar NMD mechanism in fish, as highlighted in immune cells producing Ig and TCR transcripts (44).

### Stat2 knockout affects type I but not type II IFN signaling pathway

The initial characterization of the type I IFN–induction phenotype by QPCR after incubation with recombinant type I IFN showed a general disruption of ISGs induction in the GS2 cell line. This was confirmed by the overall transcriptome analysis by RNA-seq.

In a human fibrosarcoma cell line, IRF 1 (*irf1*) has been found to be induced substantially only by type II IFN (45),and a strong modulation of this transcript is now considered as a hallmark of Stat1 dimer signaling/GAS-specific ISG (46). Our results showed that fish *irf1* gene was very strongly induced by IFNG1 but not by IFNA2 in the parental cell line EC, which supported that *irf1* is a good marker of the type II IFN response as in mammals. In GS2 cell lines, *irf1* gene was strongly induced as in the parental cell line, indicating that irf1 induction does not rely on *stat2* signaling. Similar results were reported in primary embryonic fibroblast cells isolated from stat2 knockout mice (47). In contrast, the GS1A cell line with deficient *stat1a1* and *stat1a2* genes had both IFNA2 and IFNG1 pathways disrupted. This was particularly clear with *irf1*, strongly induced after stimulation with IFNG1 in EC but not in GS1A (Fig. 2C, 2D), confirming that the signaling for IFNA2 is impacted by Stat1A proteins.

*pkr*, *mx*, or *dhx58* are type I IFN–specific ISGs (48), and their induction by IFNA2 was completely abolished in the GS2 cell line in comparison with the parental EC cell line. Similarly, primary kidney cells isolated from *stat2* knockout hamsters were unable to induce *pkr* after incubation with IFN-α (49).

These results demonstrate that in salmonid cells, Stat2 is involved essentially in the signaling pathway of type I IFN in agreement with the canonical mammalian model for the IFN signaling (50), whereas it is not required for type II IFN signaling. Thus, our results support a model in which fish type II IFN signals by Stat1:Stat1 dimers, whereas type I IFN signals by the ISGF3, a Stat2:Stat1:IRF9 heterotrimer (2). Although we observed a moderate induction of *dhx58* and *pkr* by rIFNG1 in EC cells, it was likely an indirect effect due to the modest upregulation of type I IFN by this treatment. The abolition of these effects in GS2 would therefore be consistent with the canonical model mentioned above.

Similar results were published in mammalian *stat2* knockout models such as primary culture of peritoneal macrophages harvested from *stat2* knockout mice (47) or the human U6A cell line (51). In NB4 cells (human acute promyelocytic leukemia cells), *irf1* gene is induced more intensively by type II than type I IFN (52) and follows the same pattern of expression than in EC cells.

### Viral sensitivity

Although the IFNA2 signaling pathways were completely abolished, the GS2 cell line exhibited notable elevation in the percentage viral cytopathology that was observed at the early stage of infection following infection with SPDV, EHNV, or VHSV at 15°C. This variation in the kinetics of infection between EC and GS2 cell lines may not be more remarkable than differences that would be observed between two clonal cell lines from the same origin (53, 54). However, if the viral cytopathology was mainly linked to the induction of Stat2-dependent ISGs, we would have observed a more pronounced alteration of the effect of viral infection in GS2 cells in comparison with EC cells.

The amount viral particles produced at early stage of infection with EHNV, a DNA virus, is higher in GS2 than in EC and is coherent with an increase of cytopathology with this virus. However, the results are different with VHSV, a negative ssRNA virus, whereby GS2 cells produce less viral particles. Replication of EHNV was found to be very sensitive to the level of ISG15 in cyprinid cells (55) and only mildly to the presence of Mx1 protein in salmonid cells (25). The absence of induction of these genes in GS2 may therefore explain, although partially, the increased viral cytopathy and shedding in the cell line.

This contrasted with results in mice where infection with vesicular stomatitis virus of primary or immortalized fibroblast cultures isolated from *stat2* knockout mice exhibited viral titers increased up 80- and 20-fold relative to wild type, respectively (47).

From the RNA-seq data, following stimulation with IFNA2, a discrete number of ISGs were induced significantly in both EC and GS2 cell lines. As an example, two of them, guanine nucleotide-binding protein G subunit α-like (GNAI) and glutathione peroxidase 1 (GPX-1), were induced over 2-fold in both cell lines after stimulation with IFNA2. The latter have been associated with viral sensitivity (56) and may explain, at least partially, the GS2 viral resistance. Further functional characterization of such genes may reveal important roles in the Stat2-independent antiviral activity.

### Possible role of IRF1-regulated genes in antiviral resistance at the cellular level

The induction of ISGs via alternative pathway(s) can be possible without the involvement of either Stat1 or Stat2 as described in West Nile virus Eg101–infected mouse cells (57). These results are in agreement a previous study (58) whereby surviving patients with deficient type I IFN pathway caused by Stat2 null phenotype are remarkably healthy with no evident impairment in their innate immunity. However, other reports may suggest different consequences of disappearance of Stat2 in other species. Approximately 40% of hamsters lacking Stat2 succumbs to Zika virus (59); however, in this study, there was no side-by-side comparison with the wild type.

In human, Schoggins et al. (4) reported that IRF1 inhibited many viruses, including hepatitis C virus, HIV-1, yellow fever virus, West Nile virus, Venezuelan equine encephalitis virus, and chikungunya virus, even in a *stat1*^−/−^ background, indicating that this transcription factor triggers a particular antiviral pathway. In fact, IRF1 overexpression in Stat1^−/−^ fibroblasts upregulated the expression of many well-known ISGs and effector proteins such as Mx1, IFI-6, -27, -30, -35, and 44, IFIT1 and 3, and ISG15, IRF9, SAMD9, USP18, and ISG20 but not type I IFN. Thus, the repertoire of IRF1 induced genes only partly overlaps the typical set of ISGs induced via IFNR and Jak/Stat.

Overall, our QPCR and whole transcriptome data suggest that the *irf1* expression in GS2 is not sufficient to rescue a detectable induction of ISGs. Thus, a number of fish orthologs of ISGs inducible by IRF1 overexpression in human Stat1^−/−^ fibroblasts were induced by trout IFNA2 in EC but not in GS2. These genes included *mx1*, *ifi27*, *ifi44*, *gbp1*, *lgals3BP*, *parp14*, *usp18*, *samd9l*, *dhx58*, *ifit5*, and *zc3hav1*. In fact, no gene significantly induced by type I IFN in GS2 was orthologous to a member of the IRF1-stimulated gene list reported previously (60). Overall, these results indicate that the faint (and nonsignificant) *irf1* induction observed in GS2 after type I IFN stimulation does not complement the disruption of the Stat2 dependent signaling.

We have demonstrated that the function of Stat2 in salmonid cells follows the canonical signaling pathway described in higher vertebrates. However, the effect of the Stat2 loss of function on the ability to resist to a viral infection depends on the type of virus. Further transcriptomics studies on the GS2 cell line upon early infection with different categories of viruses are required to identify genes responsible for viral resistance.

## Supplementary Material

Data Supplement

## References

[1] Sadler A. J. Williams B. R. G. 2008 Interferon-inducible antiviral effectors. Nat. Rev. Immunol. 8: 559–568. 18575461 10.1038/nri2314 PMC2522268

[2] Schneider W. M. Chevillotte M. D. Rice C. M. 2014 Interferon-stimulated genes: a complex web of host defenses. Annu. Rev. Immunol. 32: 513–545. 24555472 10.1146/annurev-immunol-032713-120231 PMC4313732

[3] Ivashkiv L. B. Donlin L. T. 2014 Regulation of type I interferon responses. Nat. Rev. Immunol. 14: 36–49. 24362405 10.1038/nri3581 PMC4084561

[4] Schoggins J. W. Wilson S. J. Panis M. Murphy M. Y. Jones C. T. Bieniasz P. Rice C. M. 2011 A diverse range of gene products are effectors of the type I interferon antiviral response. Nature 472: 481–485. 21478870 10.1038/nature09907 PMC3409588

[5] Olagnier D. Hiscott J. 2014 Type I and type III interferon-induced immune response: it’s a matter of kinetics and magnitude. Hepatology 59: 1225–1228. 24677190 10.1002/hep.26959

[6] Shi J. Zhang Y.-B. Zhang J.-S. Gui J.-F. 2013 Expression regulation of zebrafish interferon regulatory factor 9 by promoter analysis. Dev. Comp. Immunol. 41: 534–543. 23916490 10.1016/j.dci.2013.07.017

[7] Castro R. Martin S. A. M. Bird S. Lamas J. Secombes C. J. 2008 Characterisation of gamma-interferon responsive promoters in fish. Mol. Immunol. 45: 3454–3462. 18457879 10.1016/j.molimm.2008.03.015

[8] Collins C. Ganne G. Collet B. 2014 Isolation and activity of the promoters for STAT1 and 2 in Atlantic salmon *Salmo salar.* Fish Shellfish Immunol. 40: 644–647. 25128593 10.1016/j.fsi.2014.07.025

[9] Volff J. N. 2005 Genome evolution and biodiversity in teleost fish. Heredity (Edinb) 94: 280–294. 15674378 10.1038/sj.hdy.6800635

[10] Pasquier J. Cabau C. Nguyen T. Jouanno E. Severac D. Braasch I. Journot L. Pontarotti P. Klopp C. Postlethwait J. H. 2016 Gene evolution and gene expression after whole genome duplication in fish: the PhyloFish database. BMC Genomics 17: 368. 27189481 10.1186/s12864-016-2709-z PMC4870732

[11] Lien S. Koop B. F. Sandve S. R. Miller J. R. Kent M. P. Nome T. Hvidsten T. R. Leong J. S. Minkley D. R. Zimin A. 2016 The Atlantic salmon genome provides insights into rediploidization. Nature 533: 200–205. 27088604 10.1038/nature17164 PMC8127823

[12] Berthelot C. Brunet F. Chalopin D. Juanchich A. Bernard M. Noël B. Bento P. Da Silva C. Labadie K. Alberti A. 2014 The rainbow trout genome provides novel insights into evolution after whole-genome duplication in vertebrates. Nat. Commun. 5: 3657. 24755649 10.1038/ncomms4657 PMC4071752

[13] Macqueen D. J. Johnston I. A. 2014 A well-constrained estimate for the timing of the salmonid whole genome duplication reveals major decoupling from species diversification. Proc. Biol. Sci. 281: 20132881. 24452024 10.1098/rspb.2013.2881 PMC3906940

[14] Boudinot P. Langevin C. Secombes C. J. Levraud J.-P. 2016 The peculiar characteristics of fish type I interferons. Viruses 8: 298. 10.3390/v8110298 PMC5127012 27827855

[15] Briolat V. Jouneau L. Carvalho R. Palha N. Langevin C. Herbomel P. Schwartz O. Spaink H. P. Levraud J.-P. Boudinot P. 2014 Contrasted innate responses to two viruses in zebrafish: insights into the ancestral repertoire of vertebrate IFN-stimulated genes. J. Immunol. 192: 4328–4341. 24683187 10.4049/jimmunol.1302611

[16] Zou J. Tafalla C. Truckle J. Secombes C. J. 2007 Identification of a second group of type I IFNs in fish sheds light on IFN evolution in vertebrates. J. Immunol. 179: 3859–3871. 17785823 10.4049/jimmunol.179.6.3859

[17] Ng C. T. Mendoza J. L. Garcia K. C. Oldstone M. B. A. 2016 Alpha and beta type 1 interferon signaling: passage for diverse biologic outcomes. Cell 164: 349–352. 26824652 10.1016/j.cell.2015.12.027 PMC4733246

[18] Aggad D. Mazel M. Boudinot P. Mogensen K. E. Hamming O. J. Hartmann R. Kotenko S. Herbomel P. Lutfalla G. Levraud J.-P. 2009 The two groups of zebrafish virus-induced interferons signal via distinct receptors with specific and shared chains. J. Immunol. 183: 3924–3931. 19717522 10.4049/jimmunol.0901495

[19] Levraud J.-P. Boudinot P. Colin I. Benmansour A. Peyrieras N. Herbomel P. Lutfalla G. 2007 Identification of the zebrafish IFN receptor: implications for the origin of the vertebrate IFN system. J. Immunol. 178: 4385–4394. 17371995 10.4049/jimmunol.178.7.4385

[20] Secombes C. J. Zou J. 2017 Evolution of interferons and interferon receptors. Front. Immunol. 8: 209. 28303139 10.3389/fimmu.2017.00209 PMC5332411

[21] Dehler C. E. Boudinot P. Martin S. A. M. Collet B. 2016 Development of an efficient genome editing method by CRISPR/Cas9 in a fish cell line. Mar. Biotechnol. (NY) 18: 449–452. 27236514 10.1007/s10126-016-9708-6 PMC5007268

[22] Christensen K. A. Leong J. S. Sakhrani D. Biagi C. A. Minkley D. R. Withler R. E. Rondeau E. B. Koop B. F. Devlin R. H. 2018 Chinook salmon (*Oncorhynchus tshawytscha*) genome and transcriptome. PLoS One 13: e0195461. 29621340 10.1371/journal.pone.0195461 PMC5886536

[23] Zou J. Carrington A. Collet B. Dijkstra J. M. Yoshiura Y. Bols N. Secombes C. 2005 Identification and bioactivities of IFN-γ in rainbow trout *Oncorhynchus mykiss*: the first Th1-type cytokine characterized functionally in fish. J. Immunol. 175: 2484–2494. 16081820 10.4049/jimmunol.175.4.2484

[24] Collet B. Boudinot P. Benmansour A. Secombes C. J. 2004 An Mx1 promoter-reporter system to study interferon pathways in rainbow trout. Dev. Comp. Immunol. 28: 793–801. 15043947 10.1016/j.dci.2003.12.005

[25] Lester K. Hall M. Urquhart K. Gahlawat S. Collet B. 2012 Development of an in vitro system to measure the sensitivity to the antiviral Mx protein of fish viruses. J. Virol. Methods 182: 1–8. 22405879 10.1016/j.jviromet.2012.01.014

[26] Langdon J. S. Humphrey J. D. Williams L. M. 1988 Outbreaks of an EHNV‐like iridovirus in cultured rainbow trout, *Salmo gairdneri* Richardson, in Australia. J. Fish Dis. 11: 93–96.

[27] R Core Team. 2017. R: a language and environment for statistical computing. R Foundation for Statistical Computing, Vienna, Austria. Available at: https://www.R-project.org/. Accessed February 6, 2018.

[28] Oliveros, J. C. 2015. Venny. An interactive tool for comparing lists with Venn's diagrams. 2007-2015. Available at: http://bioinfogp.cnb.csic.es/tools/venny/. Accessed February 6, 2018.

[29] Trapnell C. Pachter L. Salzberg S. L. 2009 TopHat: discovering splice junctions with RNA-Seq. Bioinformatics 25: 1105–1111. 19289445 10.1093/bioinformatics/btp120 PMC2672628

[30] Liao Y. Smyth G. K. Shi W. 2014 featureCounts: an efficient general purpose program for assigning sequence reads to genomic features. Bioinformatics 30: 923–930. 24227677 10.1093/bioinformatics/btt656

[31] Love M. I. Huber W. Anders S. 2014 Moderated estimation of fold change and dispersion for RNA-seq data with DESeq2. Genome Biol. 15: 550. 25516281 10.1186/s13059-014-0550-8 PMC4302049

[32] Sobhkhez M. Skjesol A. Thomassen E. Tollersrud L. G. Iliev D. B. Sun B. Robertsen B. Jørgensen J. B. 2014 Structural and functional characterization of salmon STAT1, STAT2 and IRF9 homologs sheds light on interferon signaling in teleosts. FEBS Open Bio 4: 858–871. 10.1016/j.fob.2014.09.007 PMC4215117 25379383

[33] Rusinova I. Forster S. Yu S. Kannan A. Masse M. Cumming H. Chapman R. Hertzog P. J. 2013 Interferome v2.0: an updated database of annotated interferon-regulated genes. Nucleic Acids Res. 41(Database issue): D1040–D1046. 23203888 10.1093/nar/gks1215 PMC3531205

[34] Wang H. Yang H. Shivalila C. S. Dawlaty M. M. Cheng A. W. Zhang F. Jaenisch R. 2013 One-step generation of mice carrying mutations in multiple genes by CRISPR/Cas-mediated genome engineering. Cell 153: 910–918. 23643243 10.1016/j.cell.2013.04.025 PMC3969854

[35] Bressan R. B. Dewari P. S. Kalantzaki M. Gangoso E. Matjusaitis M. Garcia-Diaz C. Blin C. Grant V. Bulstrode H. Gogolok S. 2017 Efficient CRISPR/Cas9-assisted gene targeting enables rapid and precise genetic manipulation of mammalian neural stem cells. Development 144: 635–648. 28096221 10.1242/dev.140855 PMC5312033

[36] Jao L.-E. Wente S. R. Chen W. 2013 Efficient multiplex biallelic zebrafish genome editing using a CRISPR nuclease system. Proc. Natl. Acad. Sci. USA 110: 13904–13909. 23918387 10.1073/pnas.1308335110 PMC3752207

[37] Zu Y. Zhang X. Ren J. Dong X. Zhu Z. Jia L. Zhang Q. Li W. 2016 Biallelic editing of a lamprey genome using the CRISPR/Cas9 system. Sci. Rep. 6: 23496. 27005311 10.1038/srep23496 PMC4804306

[38] Sawamura R. Osafune N. Murakami T. Furukawa F. Kitano T. 2017 Generation of biallelic F0 mutants in medaka using the CRISPR/Cas9 system. Genes Cells 22: 756–763. 28707405 10.1111/gtc.12511

[39] Edvardsen R. B. Leininger S. Kleppe L. Skaftnesmo K. O. Wargelius A. 2014 Targeted mutagenesis in Atlantic salmon (*Salmo salar* L.) using the CRISPR/Cas9 system induces complete knockout individuals in the F0 generation. PLoS One 9: e108622. 25254960 10.1371/journal.pone.0108622 PMC4177897

[40] Lykke-Andersen S. Jensen T. H. 2015 Nonsense-mediated mRNA decay: an intricate machinery that shapes transcriptomes. Nat. Rev. Mol. Cell Biol. 16: 665–677. 26397022 10.1038/nrm4063

[41] Silva A. L. Ribeiro P. Inácio A. Liebhaber S. A. Romão L. 2008 Proximity of the poly(A)-binding protein to a premature termination codon inhibits mammalian nonsense-mediated mRNA decay. RNA 14: 563–576. 18230761 10.1261/rna.815108 PMC2248256

[42] Brogna S. Wen J. 2009 Nonsense-mediated mRNA decay (NMD) mechanisms. Nat. Struct. Mol. Biol. 16: 107–113. 19190664 10.1038/nsmb.1550

[43] Amrani N. Ganesan R. Kervestin S. Mangus D. A. Ghosh S. Jacobson A. 2004 A faux 3′-UTR promotes aberrant termination and triggers nonsense-mediated mRNA decay. Nature 432: 112–118. 15525991 10.1038/nature03060

[44] Quiniou S. M. A. A. Wilson M. Boudinot P. 2011 Processing of fish Ig heavy chain transcripts: diverse splicing patterns and unusual nonsense mediated decay. Dev. Comp. Immunol. 35: 949–958. 21168434 10.1016/j.dci.2010.12.007

[45] Der S. D. Zhou A. Williams B. R. G. Silverman R. H. 1998 Identification of genes differentially regulated by interferon alpha, beta, or gamma using oligonucleotide arrays. Proc. Natl. Acad. Sci. USA 95: 15623–15628. 9861020 10.1073/pnas.95.26.15623 PMC28094

[46] Ramsauer K. Farlik M. Zupkovitz G. Seiser C. Kröger A. Hauser H. Decker T. 2007 Distinct modes of action applied by transcription factors STAT1 and IRF1 to initiate transcription of the IFN-gamma-inducible gbp2 gene. Proc. Natl. Acad. Sci. USA 104: 2849–2854. 17293456 10.1073/pnas.0610944104 PMC1815270

[47] Park C. Li S. Cha E. Schindler C. 2000 Immune response in Stat2 knockout mice. Immunity 13: 795–804. 11163195 10.1016/s1074-7613(00)00077-7

[48] Shaw A. E. Hughes J. Gu Q. Behdenna A. Singer J. B. Dennis T. Orton R. J. Varela M. Gifford R. J. Wilson S. J. Palmarini M. 2017 Fundamental properties of the mammalian innate immune system revealed by multispecies comparison of type I interferon responses. PLoS Biol. 15: e2004086. 29253856 10.1371/journal.pbio.2004086 PMC5747502

[49] Toth K. Lee S. R. Ying B. Spencer J. F. Tollefson A. E. Sagartz J. E. Kong I. K. Wang Z. Wold W. S. M. 2015 STAT2 knockout Syrian hamsters support enhanced replication and pathogenicity of human adenovirus, revealing an important role of type I interferon response in viral control. [Published erratum appears in 2016 *PLoS Pathog*. 12: e1005392.] PLoS Pathog. 11: e1005084. 26291525 10.1371/journal.ppat.1005084 PMC4546297

[50] Majoros A. Platanitis E. Kernbauer-Hölzl E. Rosebrock F. Müller M. Decker T. 2017 Canonical and non-canonical aspects of JAK-STAT signaling: lessons from interferons for cytokine responses. Front. Immunol. 8: 29. 28184222 10.3389/fimmu.2017.00029 PMC5266721

[51] Leung S. Qureshi S. A. Kerr I. M. Darnell J. E. Jr. Stark G. R. 1995 Role of STAT2 in the alpha interferon signaling pathway. Mol. Cell. Biol. 15: 1312–1317. 7532278 10.1128/mcb.15.3.1312 PMC230354

[52] Matikainen S. Ronni T. Hurme M. Pine R. Julkunen I. 1996 Retinoic acid activates interferon regulatory factor-1 gene expression in myeloid cells. Blood 88: 114–123. 8704165

[53] Sarver N. Stollar V. 1977 Sindbis virus-induced cytopathic effect in clones of *Aedes albopictus* (Singh) cells. Virology 80: 390–400. 196393 10.1016/s0042-6822(77)80014-7

[54] Iwamoto T. Nakai T. Mori K. Arimoto M. Furusawa I. 2000 Cloning of the fish cell line SSN-1 for piscine nodaviruses. Dis. Aquat. Organ. 43: 81–89. 11145456 10.3354/dao043081

[55] Langevin C. van der Aa L. M. Houel A. Torhy C. Briolat V. Lunazzi A. Harmache A. Bremont M. Levraud J.-P. Boudinot P. 2013 Zebrafish ISG15 exerts a strong antiviral activity against RNA and DNA viruses and regulates the interferon response. J. Virol. 87: 10025–10036. 23824820 10.1128/JVI.01294-12 PMC3753986

[56] Beck M. A. Esworthy R. S. Ho Y. S. Chu F. F. 1998 Glutathione peroxidase protects mice from viral-induced myocarditis. FASEB J. 12: 1143–1149. 9737717 10.1096/fasebj.12.12.1143

[57] Pulit-Penaloza J. A. Scherbik S. V. Brinton M. A. 2012 Type 1 IFN-independent activation of a subset of interferon stimulated genes in West Nile virus Eg101-infected mouse cells. Virology 425: 82–94. 22305622 10.1016/j.virol.2012.01.006 PMC3288888

[58] Hambleton S. Goodbourn S. Young D. F. Dickinson P. Mohamad S. M. B. Valappil M. McGovern N. Cant A. J. Hackett S. J. Ghazal P. 2013 STAT2 deficiency and susceptibility to viral illness in humans. Proc. Natl. Acad. Sci. USA 110: 3053–3058. 23391734 10.1073/pnas.1220098110 PMC3581986

[59] Siddharthan V. Van Wettere A. J. Li R. Miao J. Wang Z. Morrey J. D. Julander J. G. 2017 Zika virus infection of adult and fetal STAT2 knock-out hamsters. Virology 507: 89–95. 28431283 10.1016/j.virol.2017.04.013

[60] Frontini M. Vijayakumar M. Garvin A. Clarke N. 2009 A ChIP-chip approach reveals a novel role for transcription factor IRF1 in the DNA damage response. Nucleic Acids Res. 37: 1073–1085. 19129219 10.1093/nar/gkn1051 PMC2651779

