# Supplementary material for: Viral Resistance and IFN Signaling in STAT2 Knockout Fish Cells
Source: J Immunol. 2019 May 29;203(2):465–75. doi: 10.4049/jimmunol.1801376 (PMC6612602; doi:10.4049/jimmunol.1801376)
Supplement: Data Supplement [file JI_1801376.zip › JI_1801376_Supplemental_Figures_1.pdf]

A

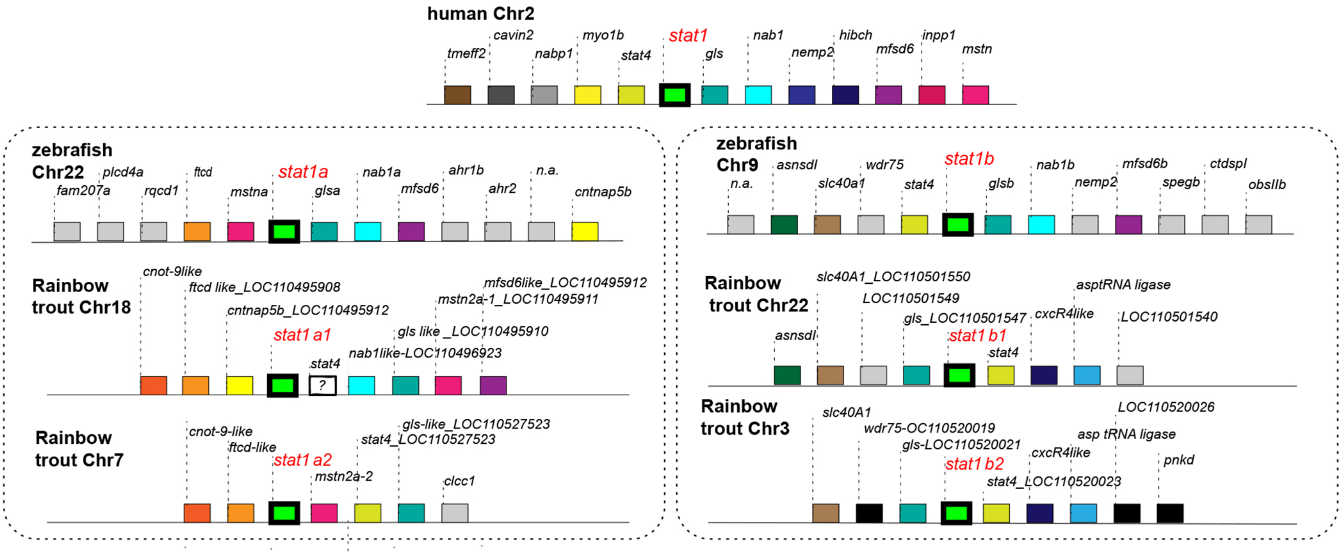

B

Locus *stat1a1* (null mutation - homozygous)

```
EC      ATG GCC CAG TGG TGC CAG CTG CAA CTA CTG GAG TCG AAA TAC CTG GAG CAG GTC GAC CAG CTC TAC GAT GAC TCC /…/
         M   A   Q   W   C   Q   L   Q   L   L   E   S   K   Y   L   E   Q   V   D   Q   L   Y   D   D   S
GS1A    ATG GCC CAG TGG TGC CAG CTG CAA -TA CTG GAG TCG --- -AC CTG GAG CAG GTC GAC CAG CTC TAC GAT GAC TCC /…/
         M   A   Q   W   C   Q   L   Q    Y   W   S   R        P   G   A   G   R   P   A   L   R   -
                ############################
                           sgRNA1          ########################
                                                     sgRNA2
```

Locus *stat1a2* (null mutations - heterozygous)

```
EC     ATG GCC CAG TGG TGC CAG CTG CAA CTA CTG GAG TCT AAA TAC CTG GAG CAG GTC GAC CAG CTC TAC GAT GAC TCC /…/
        M   A   Q   W   C   Q   L   Q   L   L   E   S   K   Y   L   E   Q   V   D   Q   L   Y   D   D   S  /…/
GS1A   ATG GCC CAG TGG TGC CAG CTG CAA C-A CTG GAG TCT AAA TAC CTG GAG CAG GTC GAC CAG CTC TAC GAT GAC TCC
        M   A   Q   W   C   Q   L   Q    H   W   S   L   N   T   W   S   K   S   T   R   S   M   M   T   P
GS1A   ATG GCC CAG TGG TGC CAG CTG CA- -TA CTG GAG TCT AAA TAC CTG GAG CAG GTC GAC CAG CTC TAC GAT GAC TCC
        M   A   Q   W   C   Q   L   H       T   G   V   -
              ############################
                         sgRNA1          ######################
                                                   sgRNA2
```

Locus *stat1b1* (not mutated)

```
EC     ATG GCC CAG TGG TTC CAG CTG CAG CAG CTA GAC TCC AAG TAC CTG GAG CAG GTG GAC CAG CTG TAT GAT GAT AAT TTC /…/
        M   A   Q   W   F   Q   L   Q   Q   L   D   S   K   Y   L   E   Q   V   D   Q   L   Y   D   D   N   F
GSA1   ATG GCC CAG TGG TTC CAG CTG CAG CAG CTA GAC TCC AAG TAC CTG GAG CAG GTG GAC CAG CTG TAT GAT GAT AAT TTC /…/
        M   A   Q   W   F   Q   L   Q   Q   L   D   S   K   Y   L   E   Q   V   D   Q   L   Y   D   D   N   F
```

Locus *stat1b2* (not mutated)

```
EC     ATG GCC CAG TGG ATC CAG CTG CAG AAA TTA GAC TCC AAG TAC CTG GAG CAG TTG GAC CAG CTG TAT GAT GAT ACT TTC /…/
        M   A   Q   W   I   Q   L   Q   K   L   D   S   K   Y   L   E   Q   L   D   Q   L   Y   D   D   T   F
GSA1   ATG GCC CAG TGG ATC CAG CTG CAG AAA TTA GAC TCC AAG TAC CTG GAG CAG TTG GAC CAG CTG TAT GAT GAT ACT TTC /…/
        M   A   Q   W   I   Q   L   Q   K   L   D   S   K   Y   L   E   Q   L   D   Q   L   Y   D   D   T   F
```

Figure S1. Isolation of *stat1a* knock out (GS1A) cell line.
The GS1A line was established following the same protocol as GS2, from the EC cell line that stably expresses both green fluorescence protein (GFP) and Cas9 (CHSE-EC). Sequences of the two highly similar paralogs stat1a1 and stat1a2 from chinook salmon (A,B) were targeted. The sequence of the sgRNAs used for mutagenesis are indicated in panel C. Following stat1a- and egfp-sgRNAs transfections, non-fluorescent clones (*i.e.*, mutants in EGFP) were chosen for characterisation, propagated and characterized. The mutations in stat1a1 and stat1a2 loci are indicated in green in panel C, while the stat1b genes were not mutated.

A. Synteny analysis of stat1 genes was performed from the rainbow trout genome, for which the assembly and annotation are more advanced compared to the newly released genome of chinook salmon.

B. Genomic characterisation of the *stat1a* loci in GS1. The location of sgRNAs is shown. The start codon is in red, exonic sequences in upper case, the protospacers are in cyan, and mutations are highlighted in green

A

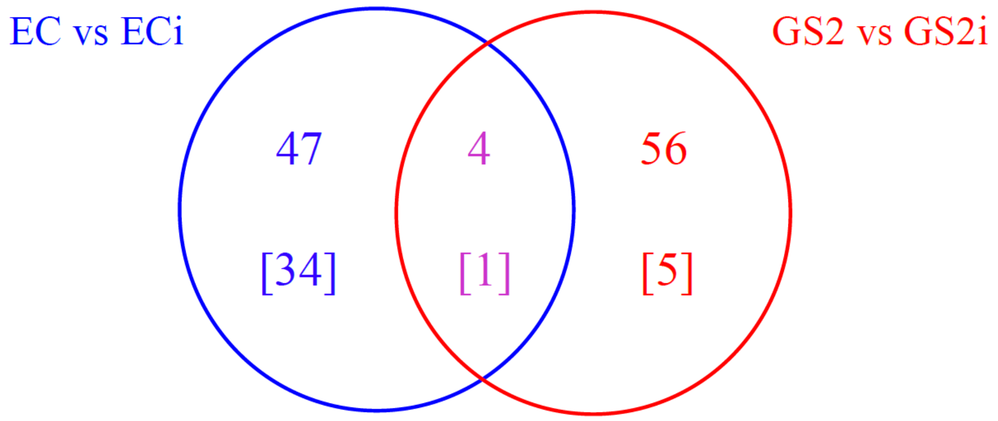

B

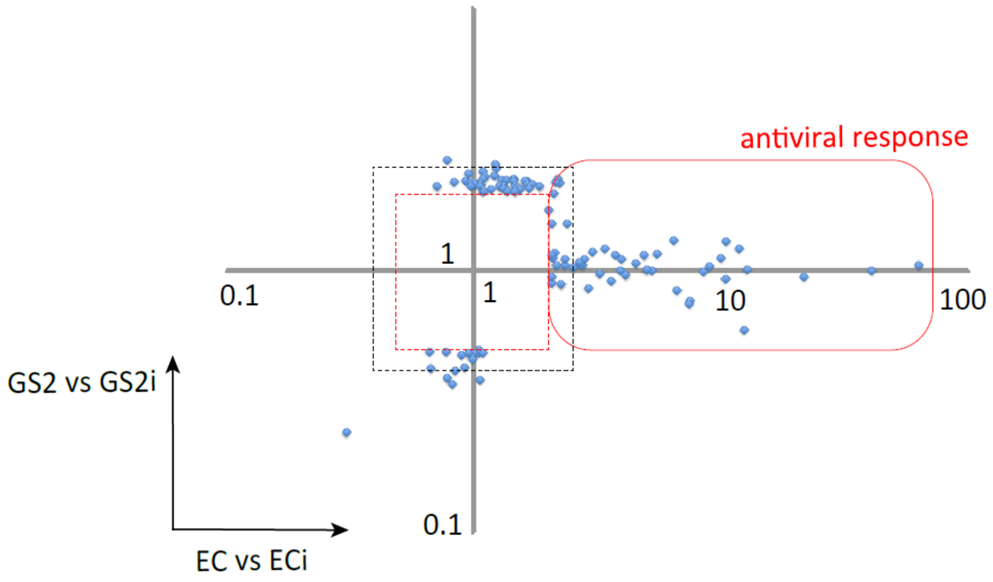

C

EC vs EC IFNA2 stimulated

Number of differential genes (adj p val < 5%; FC>2 or FC < 0.5): N=51, 50 induced, 1 repressed
David pathway analysis: GOTERM_BP_DIRECT defense response to virus adj pval=1.5 e-4

GS2 vs GS2 IFNA2 stimulated

Number of differential genes (adj p val < 5%; FC>2 or FC < 0.5): N=60, 45 induced, 15 repressed
David pathway analysis: GOTERM_BP_DIRECT Platelet aggregation adj pval=2.4 e-3
KEGG_PATHWAY Regulation of actin cytoskeleton p=3.6 e-3 (based on the same genes)

Figure S2. Transcriptome compared analysis of IFNA2 stimulation of EC and GS2 cell lines. (A) Venn diagram of differentially expressed in EC or GS2 after IFNA2 stimulation (adj p val < 0.05; FC>2 or FC < 0.5); gene numbers for (adj p val < 0.05; FC>2.5 or FC < 0.4) are shown between [ ]. (B) Log FC representation of EC and GS2 transcriptional responses to IFNA2 stimulation FC/FC representation of genes differentially expressed in one of the cell lines (adj p val < 0.05; FC>2 or FC < 0.5). The red line highlights the group of genes induced in EC (but not in GS2), mainly ISGs. Genes with 0.5<FC<2 are not represented. FC thresholds of 2/0.5 and 2.5/0.4 are represented by red and black dotted lines respectively. (C) Gene Ontology analysis of genes modulated by IFNA2 in EC and GS2 cells.
